# Theanine Improves High-Dose Epigallocatechin-3-Gallate-Induced Lifespan Reduction in *Caenorhabditis elegans*

**DOI:** 10.3390/foods10061404

**Published:** 2021-06-17

**Authors:** Yuxuan Peng, Shen Dai, Yan Lu, Ligui Xiong, Jianan Huang, Zhonghua Liu, Yushun Gong

**Affiliations:** 1 National Research Center of Engineering and Technology for Utilization of Botanical Functional Ingredients from Botanicals, Hunan Agricultural University, Changsha 410128, China; m15973781166@163.com (Y.P.); luyan900@163.com (Y.L.); xiongligui@sina.com (L.X.); 2 College of Physical Education, Hunan City University, Yiyang 413002, China; 3 Key Laboratory of Tea Science of Ministry of Education, Hunan Agricultural University, Changsha 410128, China; daishen@cofco.com (S.D.); jian7513@sina.com (J.H.); 4 Collaborative Innovation Center of Utilization of Functional Ingredients from Botanicals, Hunan Agricultural University, Changsha 410128, China

**Keywords:** theanine, EGCG, hormesis, Reactive Oxygen Species (ROS), DAF-16

## Abstract

Epigallocatechin-3-gallate (EGCG) is the most abundant polyphenol in green tea. Our previous report showed that induced hormesis was a critical determinant for the promotion of a healthy lifespan in *Caenorhabditis elegans*. In the present study, we investigated the anti-aging effects of the main active ingredients in green tea. We found that galloylated catechins (EGCG and epicatechin gallate) could extend the lifespan of *C. elegans*, while their metabolites (gallic acid, epicatechin, and epigallocatechin) could not. Interestingly, the combination with theanine, not caffeine, could alleviate the adverse effects induced by high-dose EGCG, including the promotion of lifespan and locomotor ability. This was due to the attenuation of the excess production of reactive oxygen species and the activation of DAF-16. These findings will facilitate further studies on the health benefits of tea active components and their interactions.

## 1. Introduction

Tea is one of the most popular beverages worldwide [1] and is known for its beneficial effects on human health [2,3]. Its typical contribution to health benefits mainly lies in tea polyphenols [4], including epigallocatechin-3-gallate (EGCG), epicatechin (EC), epigallocatechin (EGC) and epicatechin gallate (ECG) [5].

EGCG, the major polyphenol present in tea, mediates many known biological benefits [6]. It promotes a healthy lifespan in *Caenorhabditis elegans* and *Drosophila melanogaster* in an inverted U-shaped biphasic manner [7,8,9]. However, excess use of EGCG supplements can lead to a variety of harmful consequences [10], shorten the lifespan of *C. elegans* and *Drosophila*, and cause liver damage in humans and rodents [9,11,12,13]. How EGCG acts as a health-promoting agent in tea remains largely unanswered.

Tea is a complex beverage containing many biologically active components [14]. In addition to EGCG, theanine and caffeine also have health benefits [15,16]. While theanine can improve resistance to abiotic stresses, such as paraquat, temperature, and oxidation, its life-extending effect is controversial [15,17]. The impact of caffeine on lifespan is also inconclusive, with varied results depending on the conditions, dose, and length of exposure [16,18]. To date, no systematic studies have been conducted on the life-extending action of the active components in tea. In the present study, we aimed to investigate the influence of tea active ingredients on the longevity of *C. elegans*.

## 2. Materials and Methods

### 2.1. Chemicals

EGCG, ECG, EGC, EC, caffeine (CAF), and theanine (THA), all with a purity of more than 98%, were purchased from Hunan Sanfu Biotechnology Co. (Changsha, China). Gallic acid (GA), N-acetylcysteine (NAC), and glutamine (Gln) were purchased from Sigma-Aldrich (St. Louis, MO, USA). All other chemicals were analytically pure.

### 2.2. C. elegans Strains

The strains were obtained from the Caenorhabditis Genetics Center (CGC), University of Minnesota, Minneapolis, MN: N2 (Bristol, wild type), CB1370 (*daf-2(e1370) III*), GA186 (*sod-3(tm760) X*), CF1038 (*daf-16(mu86) I*), KN259 (huIs33 [sod-3::GFP + rol-6(su1006)]2), and TJ356 (daf-16p::daf-16a/b::GFP + rol-6(su1006)], JV1 (*unc-119(ed3) III*; jrIs1). Worms were cultivated in standard nematode growth medium (NGM) on agar plates seeded with *Escherichia coli* (OP50). The bleaching method was used to prepare synchronous clusters of *C. elegans*. OP50 were all heat inactivated at 65 °C for 30 min after overnight incubation at 37 °C. Unless otherwise stated, all experiments were performed at 20 °C.

### 2.3. Lifespan Analysis

Synchronized wild-type nematodes were cultured at 20 °C for 48 h and transferred to NGM plates containing 50 μmol L^−1^ 5-fluoro-2’-deoxyuridine (FUdR). FUdR has been shown to have an influence on the lifespan and health of *C. elegans* [19]. Therefore, FUdR was not used in all other experiments unless otherwise stated. L4 larvae were transferred to 35 mm NGM plates dosed with EGCG, ECG, EGC, EC, CAF, THA, NAC, and Gln, all of which were smeared on the surface of agar. Approximately 30–35 L4 worms were transferred onto the plates. Animal survival was counted every two days from the first day of adulthood until death. Nematodes that failed to respond to contact stimuli were considered dead. All experiments were performed in a blinded manner.

### 2.4. Length Measurement

Length measurement was based on the method described by Saul et al. [20]. Day 6 adult worms of different groups were killed at high temperature (45 °C for 2.5 h), and Image Pro Plus 6.0 (IPP) software was used to measure the body length.

### 2.5. Locomotion Assays

Locomotion was determined according to the method of Brown et al. [21]. Locomotor performance of the worms was assessed by measuring the number of body bends. A single body bend was considered a complete left to right and back to the left bend. Day 6 to day 16 adult worms were suspended in M9 buffer, and the number of bends was counted for 30 s.

### 2.6. Reactive Oxygen Species (ROS) Production

Real-time H_2_O_2_ production in the JV1 strain was explored following the methodology described by Back et al. [22]. The JV1 strain genetically expresses the hydrogen peroxide-specific sensor Hyper (University of Minnesota, MN, USA). This sensor consists of a yellow fluorescent protein that, through selective and sensitive oxidation by H_2_O_2_, creates a disulfide bridge between the isolated portions of OxyR-RD and subsequently alters the fluorescent properties of the protein [22]. Six to eight adult nematodes were placed on agar cover slides containing 2% agarose solution, and 3–5 μL 0.2 mM levamisole solution was added to paralyze them. Fluorescence images were collected using a Zeiss LSM710 confocal microscope (Carl Zeiss AG, Jena, Germany), and the images were collected under the conditions of excitation wavelengths 490 (oxidation) and 405 nm (reduction), and an emission wavelength of 535 nm. The sample fluorescence was compared with the fluorescence produced by a H_2_O_2_ standard curve to calculate the concentrations of H_2_O_2_ released from the nematodes. From day 0 to day 10 of the adult nematodes, the nematodes were tested every two days, and three independent parallel experiments were designed for each treatment. The average fluorescence intensity after collection was analyzed using Fiji software (NIH, Bethesda, MD, USA) [23].

### 2.7. DAF-16::GFP Localization Assays

DAF-16 nuclear localization was based on the method described by Li et al. [24]. The fluorescence image was captured through the GFP channel using a ZEISS positive microscope. For the effect of EGCG and theanine on DAF-16::GFP localization, each worm was scored as follows: cytoplasmic, 0; weakly nuclear, 1; strongly nuclear, 2.

### 2.8. SOD-3::GFP Expression

The expression of SOD-3::GFP was determined as described by Motta et al. [25]. Six to eight adult nematodes were placed on agar cover slides containing 2% agarose solution, and 3–5 μL 0.2 mM levamisole solution was added to paralyze them. The fluorescence image was captured through the GFP channel using a ZEISS positive microscope. The average fluorescence intensity after collection was analyzed using Fiji software.

### 2.9. Statistical Analyses

All independent experiments were repeated at least 3 times. The graphs were prepared using GraphPad Prism 8 (GraphPad Software, San Diego, CA, USA) software. Statistical analysis was performed using SPSS18.0 (Demo version, Northampton, MA, USA) software. All comparisons for differences among two and more than two data sets were performed by one-way analysis of variance and Tukey’s post-hoc test. Significance was established at *p* < 0.05.

## 3. Results

### 3.1. The Ester Group Plays a Crucial Role in EGCG-Induced Longevity

**Figure 1:**
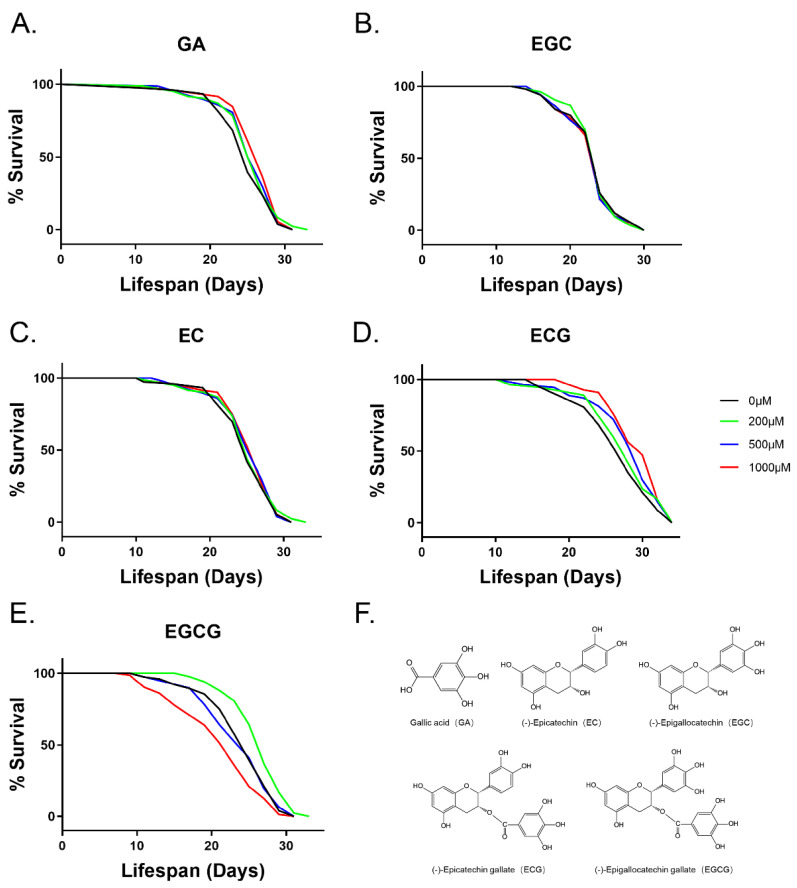
Survival of wild-type *C. elegans* treated with polyphenols. (**A**) GA, (**B**) EGC, (**C**) EC, (**D**) ECG, and (**E**) EGCG tested at different concentrations (200, 500, and 1000 μM) for their ability to change the lifespan of the worms. (**F**) Molecular formulae of GA, EGC, EC, ECG, and EGCG. Survival rates were recorded every other day until all worms died (*n* = 90–105 worms/treatment). Statistical analysis using the log-rank (Mantel–Cox) test showed that the changes in the survival curves of 500 and 1000 μM ECG compared with the control group were significant (*p* < 0.05).

In our previous studies, EGCG showed a biphasic effect on the lifespan of nematodes. The average lifespan of nematodes was maximized by 200 μM EGCG and significantly shortened by 1000 μM EGCG [9]. GA and EGC are the major metabolites of EGCG [26]. To assess the role of GA and EGC during the aging process of *C. elegans*, we treated the adults at different concentrations and measured their lifespan. We found that neither of them extended the worms’ lifespan (Figure 1A,B, Table S1). We further investigated EC and ECG, and found that ECs containing multiple phenolic hydroxyl groups could not prolong the lifespan of nematodes, while ECG containing both phenolic hydroxyl and ester groups increased longevity (Figure 1C,D,F, Table S1). Taken together, these results suggest that the ester group plays a crucial role in the anti-aging effect of EGCG.

### 3.2. Theanine Alleviates the Shortened Lifespan Induced by High-Dose EGCG in C. elegans

**Figure 2:**
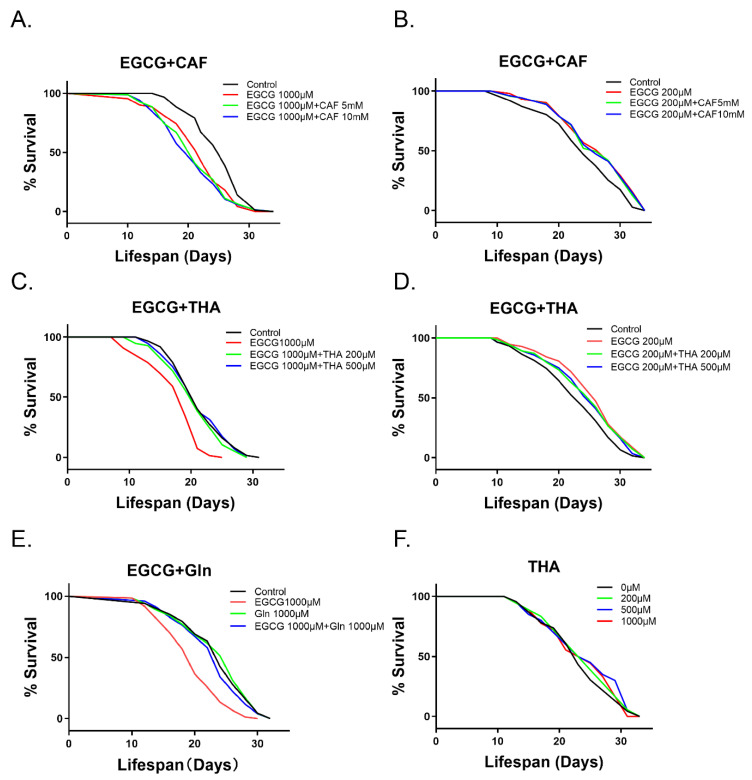
Survival of wild-type *C. elegans* treated with EGCG (200 and 1000 μM), caffeine (CAF) (5 and 10 m M), theanine (THA) (200, 500, and 1000 μM), and glutamine (Gln) (1000 μM). (**A**,**B**) Survival curves with EGCG and caffeine; (**C**,**D**) survival curves with EGCG and theanine; (**E**) survival curves with EGCG and glutamine. (**F**) Theanine was tested at different concentrations to evaluate its ability to change the lifespan of the worms. Survival was recorded every other day, until all worms died (*n* = 90–105 worms/treatment).

To study the effect of theanine and caffeine on EGCG-induced lifespan changes, we used different concentrations of theanine and caffeine, combined with high-dose EGCG (1000 μM) and low-dose EGCG (200 μM) to treat nematodes. Caffeine did not affect hormesis induced by EGCG (Figure 2A,B, Table S2). However, theanine could alleviate the shortened lifespan induced by high-dose EGCG (Figure 2C, Table S2), while it could not extend or shorten the lifespan induced by low-dose EGCG (Figure 2D, Table S2). Glutamine, a homologue of theanine, maintained a similar function (Figure 2E, Table S2). Theanine treatment alone had no effect on lifespan (Figure 2F, Table S2).

**Figure 3:**
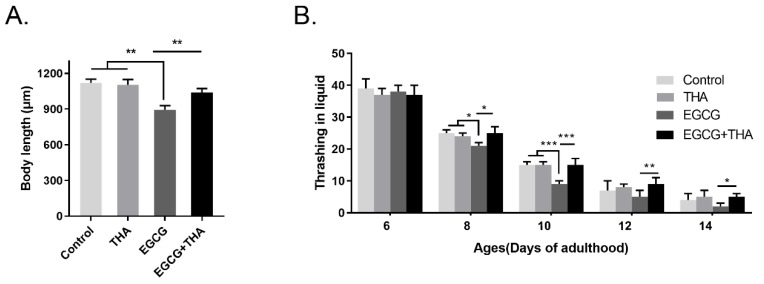
Effects of EGCG and theanine on body length and the physical exercise ability of *C. elegans*. (**A**) Nematode body length was measured on the sixth day of adulthood. The data are presented as the average of 3 trials with a total of 90–105 nematodes per concentration. (**B**) Worms were treated with EGCG (1000 μM) and theanine (200 μM) starting from adulthood (day 0). The thrashing of treated and untreated nematodes was measured on the sixth day to the fourteenth day of adulthood. All error bars represent Standard Error of Mean (SEM), and differences were considered significant at * *p* < 0.05, ** *p* < 0.01, and *** *p* < 0.001.

Body length and locomotion behavior were used as aging-associated indicators to assess the lifespan of *C. elegans*. To further verify the effect of theanine on hormesis induced by EGCG, we evaluated the nematode body lengths and bends. Theanine treatment had no effect on motility or body size. High-dose EGCG caused a significant decrease in motility and body size, while theanine showed an obvious protective effect (Figure 3A,B). Taken together, these results demonstrate that theanine improves hormesis induced by a high dose of EGCG.

### 3.3. The Dynamic Changes in ROS Levels Induced by High-Dose EGCG Were Eliminated by Theanine

**Figure 4:**
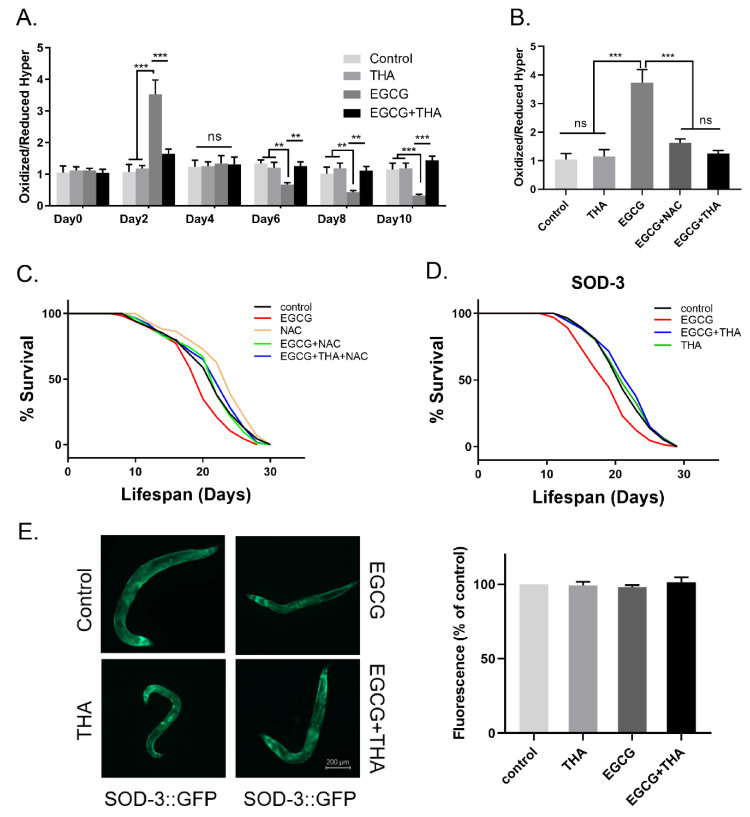
Changes in Reactive Oxygen Species (ROS) levels induced by high doses of EGCG were eliminated by theanine. (**A**) Relative formation of ROS throughout the life cycle after exposure to EGCG (1000 μM), N-acetylcysteine (NAC) (5 mM), and theanine (200 μM) in the JV1 worms. (**B**) Relative formation of ROS after 72 h of exposure to EGCG, NAC, and theanine in the JV1 worms. (**C**) Survival curves with EGCG, NAC, and theanine. (**D**) SOD-3 mutant strains were treated with EGCG and theanine starting from adulthood (day 0). Survival was recorded every two days, until all of the worms died (*n* = 90–105 worms/treatment). (**E**) SOD-3 expression after 72 h exposure to EGCG and theanine, representative images are shown (×200 magnification). All error bars represent SEM, and differences were considered significant at ** *p* < 0.01 and *** *p* < 0.001, ns—no significance.

Previous studies have shown that EGCG-induced lifespan extension of nematodes is related to ROS production [9]. Here, we found a transient increase in ROS formation after 2 days of exposure to high-dose EGCG, and a persistent decrease in ROS was observed at 8 days and beyond (Figure 4A). The addition of theanine restored the ROS levels to that of their age-matched controls (Figure 4A). To verify whether this ability of theanine was analogous to the antioxidant N-acetylcysteine (NAC), we evaluated whether the lifespan of worms treated with high-dose EGCG could be prolonged by adding 5 mM NAC. NAC abolished the EGCG-induced increase in ROS levels and prolonged the lifespan of the nematodes (Figure 4B,C, Table S3). This shows that theanine improves the lifespan of high-dose EGCG by eliminating excess ROS generation. However, theanine exhibited activity in SOD-3 mutants (GA186), which ameliorated the high dose of EGCG-induced lifespan shortening (Figure 4D, Table S3), while no effect was observed on the expression of SOD-3::GFP (Figure 4E). Theanine treatment alone had no influence on SOD-3 mutants and the expression of SOD-3::GFP. Thus, the activity of theanine in improving high-dose EGCG-induced lifespan reduction is due to the elimination of dynamic changes in ROS levels, not directly related to SOD-3.

### 3.4. DAF-16 Mediates Theanine Alleviation of High-Dose EGCG-Induced Lifespan Shortening

**Figure 5:**
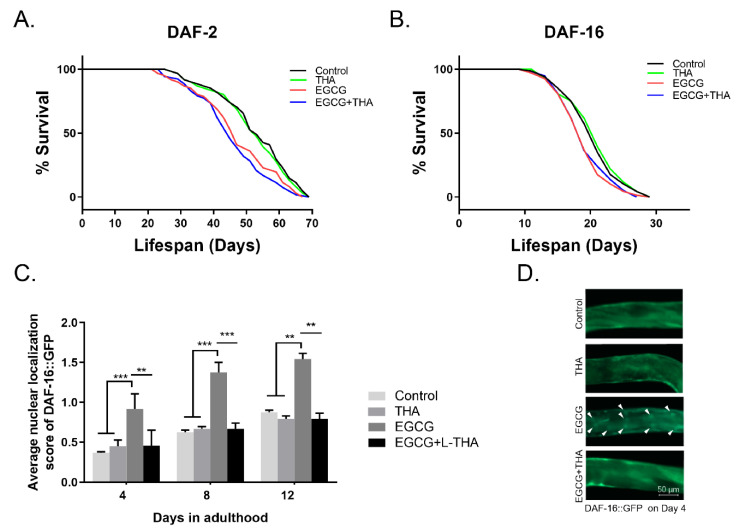
DAF-16 mediates theanine regulation of high-dose EGCG-induced lifespan shortening. (**A**,**B**) DAF-16 and DAF-2 mutant strains were treated with EGCG (1000 μM) and theanine (200 μM) starting from adulthood (day 0). Survival was recorded every two days, until all worms died (*n* = 95–105 worms/treatment). (**C**,**D**) EGCG-induced nuclear accumulation of DAF-16::GFP in N2 worms treated with EGCG and theanine. Representative images and the quantitation result are shown; the localization of DAF-16::GFP from day 4 to day 12 was compared using the Fisher exact test. All error bars represent SEM, and differences were considered significant at ** *p* <0.01 and *** *p* < 0.001.

In wild-type worms, DAF-16 translocates into the nucleus and activates the expression of genes in response to various external stimuli, such as oxidative stress, heat stress, and endoplasmic reticulum stress [24]. In our previous study [9], EGCG required DAF-16 to extend lifespan, and the role of DAF-16 may be dependent on the insulin signaling pathway. Therefore, we investigated whether DAF-2 and DAF-16 mediated the beneficial effects of theanine. The results show that theanine did not alleviate the lifespan shortening induced by high doses of EGCG in DAF-2 and DAF-16 mutants (Figure 5A,B, Table S4), suggesting that the protective effect of theanine may depend on DAF-16. We found that on adult days 4 to 12, there was substantial nuclear accumulation of DAF-16 in N2 worms grown at high-dose EGCG, but not in control nematodes. The addition of theanine slowed down the nuclear localization of DAF-16 and resulted in a growth trend in age-matched controls (Figure 5C and Table S5). This suggests that theanine alleviates the external stress pressure caused by high-dose EGCG in wild-type worms. The microscopy results (Figure 5D, Figure S1) show that the nematodes growing in EGCG increased the nuclear location of DAF-16 on day 4 of adulthood. EGCG triggered nuclear localization of DAF-16, and addition of theanine caused the nuclear DAF-16F::GFP signal to fade (Figure 5C,D). These results confirm that theanine alleviates DAF-16 nuclear accumulation induced by high-dose EGCG, thus prolonging lifespan.

## 4. Discussion

In this study, we found that the ester group is vital for the anti-aging activity of EGCG. Caffeine did not exhibit synergistic or antagonistic effects on hormesis induced by EGCG. Theanine plays a positive role in hormesis induced by EGCG and alleviates lifespan shortening by regulating dynamic ROS level changes and DAF-16 nuclear accumulation.

Many studies have reported a correlation between the natural antioxidant compounds and antiaging capacities [11,20,27]. Free radical scavenging properties are the most renowned biological actions of EGCG [28]. It depends on the hydroxyl groups bound to the aromatic ring [29]. In a previous study, we found that EGCG at lower concentrations (50–300 μM) promoted longevity [9]. In fact, EGCG has shown efficacy in *C. elegans*, *Drosophila*, and mice by extending lifespan only up to a certain dose range [8,11,12,21]. EGCG is a gallate-type catechin formed via the esterification of EGC and GA [30]. GA can extend the lifespan slightly in *C. elegans* when fed live bacteria, but not when fed dead bacteria. Antibacterial capacities are thought to be fundamental to GA-mediated lifespan extension [20]. We found that neither GA nor EGC extended the lifespan of *C. elegans* fed dead bacteria (Figure 1A,B). In addition, we found that ECG, not EC, extended the lifespan of *C. elegans* (Figure 1C,D), although many reports have shown that they both exhibit antioxidant activities in vitro [31]. Consistent with previous observations [20], the antioxidant activity of catechins does not necessarily lead to anti-aging activity. In our study, we found that the anti-aging activity of ECG was more powerful than that of the metabolites (GA, EC, and EGC). Interestingly, in terms of antibacterial properties and antibiotic sensitization, galloylated catechins (ECG and EGCG) have more significant effects than non-galloylated catechins (EC and EGC) because of their unique molecular structure [32]. This may be related to the fact that the ester group alters the membrane permeability by binding to the cell membrane to affect the transmembrane transport of substances [32,33]. Thus, it might improve the bioavailability of galloylated catechins to extend the lifespan in *C. elegans*. Therefore, unlike previous studies [29,34,35], we considered that the ester group may contribute to the health benefits of EGCG.

Caffeine and theanine were recently found to increase the absorption of EGCG [33]. In our study, theanine alleviated high-dose EGCG-induced lifespan shortening, while caffeine did not (Figure 2A,D). EGCG easily undergoes auto-oxidation under common experimental conditions [36], which can produce the EGCG semiquinone radical, which can produce EGCG quinone [37]. The EGCG quinone is electrophilic, and thus prone to react with nucleophilic groups on proteins or free amino acids [38]. Therefore, theanine may bind primarily to EGCG quinone, which decreases the concentration of EGCG, thus alleviating the high-dose EGCG-induced shortened lifespan. However, we found that the anti-aging activity of low-dose EGCG was not abolished by theanine. This phenomenon requires further investigation.

In *C. elegans*, the mechanism of life extension by 200 μM EGCG is stimulated by the induced production of ROS [39]. In a previous study, we found that the low-dose EGCG-induced lifespan extension was abolished by the administration of the antioxidant NAC [9]. In our study, the excessive increase in ROS levels induced by 1000 μM EGCG caused a similar shortening of lifespan to that induced by high concentrations of oxidants [20,21]. NAC abrogated the excessive increase in ROS levels induced by 1000 μM EGCG, thus recovering the shortened lifespan (Figure 4C). Unlike NAC, theanine reduced the level of 1000 μM EGCG-induced excessive ROS accumulation and had no influence on the lifespan extension induced by 200 μM EGCG (Figure 2D and Figure 4C). This suggests that theanine may be involved in redox regulation in *C. elegans*, and not only act as an antioxidant.

DAF-16 is a forkhead transcription factor (FoxO) involved in resistance, lifespan, and metabolism in a variety of organisms, including worms, flies, and rodents [40]. The role of DAF-16 in lifespan extension induced by EGCG may be dependent on the insulin signaling pathway [41]. In our study, high-dose EGCG shortened the lifespan of SOD-3 (Figure 4D), DAF-2-, and DAF-16-mutants (Figure 5A,B), suggesting that the lifespan reduction induced by high-dose EGCG may not be entirely dependent on the insulin receptor pathway. DAF-16 is activated during normal aging and accumulates at an accelerated rate in response to stress. In N2 worms, nuclear DAF-16 accumulates in older but not in young animals [24]. The accumulation of DAF-16 is accelerated earlier under external stress conditions such as high temperature and oxidative stress [42]. This suggests that the activation of DAF-16 might be driven by aging. High doses of EGCG triggered the nuclear localization of DAF-16 in wild-type worms and caused its aggregation in early to mid-adulthood (Figure 5C), suggesting that high doses of EGCG accelerate the aging process in worms. Theanine alleviated high-dose EGCG-induced nuclear accumulation of DAF-16 in early to mid-adulthood (Figure 5C), thereby delaying aging. Meanwhile, theanine did not alleviate the shortened lifespan of DAF-2- and DAF-16-mutants induced by high-dose EGCG (Figure 5A,B). This suggests that theanine regulated the high-dose EGCG-induced lifespan reduction through an insulin-related pathway.

According to the theory of traditional Chinese medicine, the optimal therapeutic effect can be achieved by a combination of different active substances, which is a new consideration when making use of plant functional components in medical research [43]. Different active substances may exert their therapeutic effects through the synergistic actions of multiple signaling pathways and targets to achieve more significant health benefits [44]. Our results suggest a synergistic interaction between EGCG and theanine. The combination of EGCG and theanine did not enhance anti-aging activity, while theanine effectively alleviated the high-dose EGCG-induced lifespan reduction. Theanine may mediate hormesis induced by EGCG through its affinity to ester groups. However, further studies are needed to elucidate the synergistic effect between EGCG and theanine in tea.

## 5. Conclusions

In conclusion, we reported that galloylated catechins showed more potent anti-aging activity than non-galloylated catechins. We also revealed that theanine improved the shortened lifespan induced by high doses of EGCG. The results of the present study provide novel insights into the interactions and mechanisms of the main functional components in tea.

## Supplementary Materials

The following are available online at https://www.mdpi.com/article/10.3390/foods10061404/s1, Figure S1: related to Figure 5D, Table S1: related to Figure 1A–E, Table S2: related to Figure 2A–F; Table S3: related to Figure 4C,D; Table S4: related to Figure 5A,B.
