# Supplementary material for: Theanine Improves High-Dose Epigallocatechin-3-Gallate-Induced Lifespan Reduction in Caenorhabditis elegans
Source: Foods. 2021 Jun 17;10(6):1404. doi: 10.3390/foods10061404 (PMC8235257; doi:10.3390/foods10061404)
Supplement: Supplementary file 1 [file foods-10-01404-s001.zip › foods-1230592-supplementary.pdf]

Table S1 （related to Figure 1A, 1B, 1C, 1D, 1E）

Treatment (N2/20℃)	Concen-trations (μM)	Mean Lifespan(d) ±SEM(P Value)	Median Lifespan(d) ±SEM(P Value)	N
GA	0	24.9 ±0.5 (n.s.)	25.0 ±0.4 (n.s.)	100
	50	24.7 ±0.5 (n.s.)	25.0 ±0.4 (n.s.)	95
	100	25.1 ±0.5 (n.s.)	25.0 ±0.5 (n.s.)	98
	200	25.5 ±0.4 (n.s.)	25.0 ±0.4 (n.s.)	99
	500	25.6 ±0.4 (n.s.)	25.0 ±0.4 (n.s.)	95
	1000	26.2 ±0.4 (<0.1)	27.0 ±0.5 (<0.1)	95
EGC	0	23.3 ±0.5 (n.s.)	24.0 ±0.5 (n.s.)	105
	50	22.6 ±0.5 (n.s.)	24.0 ±0.6 (n.s.)	95
	100	23.0 ±0.4 (n.s.)	24.0 ±0.4 (n.s.)	105
	200	23.5 ±0.4 (n.s.)	24.0 ±0.2 (n.s.)	105
	500	23.2 ±0.4 (n.s.)	24.0 ±0.2 (n.s.)	105
	1000	23.2 ±0.4 (n.s.)	24.0 ±0.2 (n.s.)	101
EC	0	24.3 ±0.2 (n.s.)	25.0 ±0.3 (n.s.)	105
	50	24.2 ±0.3(n.s.)	25.0 ±0.4 (n.s.)	105
	100	24.4 ±0.3 (n.s.)	25.0 ±0.4 (n.s.)	105
	200	24.3 ±0.4 (n.s.)	25.0 ±0.4 (n.s.)	105
	500	24.7 ±0.4 (n.s.)	25.0 ±0.4 (n.s.)	98
	1000	24.9 ±0.5 (n.s.)	26.0 ±0.4 (n.s.)	105
ECG	0	27.3 ±0.6 (n.s.)	28.0 ±0.8 (n.s.)	105
	50	29.1 ±0.6 (n.s.)	30.0 ±0.6 (n.s.)	96

	100	27.5 ±0.6 (n.s.)	28.0 ±0.5 (n.s.)	105
	200	27.9 ±0.8 (n.s.)	28.0 ±0.7 (n.s.)	105
	500	28.5 ±0.7 (n.s.)	30.0 ±0.6 (n.s.)	100
	1000	30.7 ±0.7 (<0.001)	32.0 ±1.2 (<0.001.)	105
EGCG	0	24.2 ±0.5 (n.s.)	25.0 ±0.6 (n.s.)	105
	50	24.8 ±0.5 (n.s.)	25.0 ±0.5 (n.s.)	105
	100	26.4 ±0.4 (<0.01)	27.0 ±0.5 (<0.01)	100
	200	26.6 ±0.4 (<0.001)	27.0 ±0.4 (<0.001)	100
	500	23.8 ±0.5 (n.s.)	25.0 ±1.0 (n.s.)	97
	1000	21.3 ±0.7 (<0.001)	23.0 ±0.8 (<0.001)	102

n.s.= not significant

N = total worm number

P value compared to the Control (0) group

Table S2 （related to Figure 2A, 2B, 2C, 2D, 2E, 2F）

Treatment（N2/20°C）	Mean Lifespan(d) ±SEM(P Value)	Median Lifespan(d) ±SEM(P Value)	N
Control	25.2 ±0.5 (n.s.)	27.0 ±0.4 (n.s.)	105
EGCG 1000μM	22.3 ±0.3 (<0.001)	23.0 ±0.6 (<0.001)	95
EGCG 1000μM +CAF 5mM	20.1 ±0.5 (<0.001)	21.0 ±0.5 (<0.001)	95
EGCG 1000μM +CAF 10mM	22.5 ±0.4 (<0.001)	24.0 ±0.4 (<0.001)	95
Control	23.5 ±0.5 (n.s.)	24.0 ±0.5 (n.s.)	105
EGCG 200μM	25.1 ±0.5 (<0.01)	26.0 ±0.4 (<0.01)	105
EGCG 200μM +CAF 5mM	25.0 ±0.4 (<0.01)	26.0 ±0.2 (<0.01)	102
EGCG 200μM +CAF 10mM	24.8 ±0.5 (<0.01)	26.0 ±0.4 (<0.01)	102
Control	21.4 ±0.5 (n.s.)	21.0 ±0.5 (n.s.)	104
EGCG 1000μM	17.5 ±0.5 (<0.001)	19.0 ±0.5 (<0.001)	95
EGCG 1000μM +THA 200μM	20.7 ±0.6 (n.s.)	21.0 ±0.8 (n.s.)	98
EGCG 1000μM +THA 500μM	21.2 ±0.6 (n.s.)	21.0 ±0.6 (n.s.)	98
Control	22.5 ±0.5(n.s.)	23.0 ±0.5 (n.s.)	104
EGCG 200μM	26.4 ±0.6 (<0.001)	26.0 ±0.5(<0.001)	100
EGCG 200μM +THA 200μM	24.7 ±0.5 (<0.001)	25.0 ±0.5 (<0.001)	105
EGCG 200μM +THA 500μM	24.6 ±0.4 (<0.001)	25.0 ±0.5(<0.001)	105
Control	23.4 ±0.7 (n.s.)	24.0 ±0.6 (n.s.)	105
EGCG 1000μM	19.7 ±0.5 (<0.001)	20.0 ±0.6 (<0.001)	95
Gln 1000μM	23.5 ±0.7 (n.s.)	26.0 ±0.7 (n.s.)	102
EGCG 1000μM +Gln 1000μM	26.6 ±0.6 (n.s.)	24.0 ±0.5 (n.s.)	103
Control	23.1 ±0.5 (n.s.)	23.0 ±0.4 (n.s.)	100

THA 200μM	23.6 ±0.4 (n.s.)	23.0 ±0.5 (n.s.)	98
THA 500μM	23.5 ±0.5 (n.s.)	24.0 ±0.6 (n.s.)	99
THA 1000μM	23.2 ±0.5 (n.s.)	23.0 ±0.5 (n.s.)	102

n.s.= not significant

N = total worm number

P value compared to the Control (0) group

Table S3 （related to Figure 4C, 4D）

Treatment	Mean Lifespan(d) ±SEM(P Value)	Median Lifespan(d) ±SEM(P Value)	N
Control（N2/20℃）	20.9 ±0.7 (n.s.)	22.0 ±0.6 (n.s.)	105
EGCG 1000μM（N2/20℃）	19.3 ±0.6 (<0.01)	20.0 ±0.6 (<0.01)	95
NAC 5mM（N2/20℃）	21.0 ±0.7(n.s.)	22.0 ±1.1 (n.s.)	95
EGCG 1000μM +NAC 5mM（N2/20℃）	21.1 ±0.6 (n.s.)	22.0 ±0.4 (n.s.)	95
EGCG 1000μM +NAC 5Mm+THA 200μM（N2/20℃）	21.3 ±0.7 (n.s.)	22.0 ±0.7 (n.s.)	102
Control（SOD-3/20℃）	21.2 ±0.5 (n.s.)	21.0 ±0.6 (n.s.)	105
EGCG 1000μM（SOD-3/20℃）	19.1 ±0.5 (<0.01)	19.0 ±0.9 (<0.01)	95
EGCG 1000μM + THA 200μM（SOD-3/20℃）	21.7 ±0.6 (n.s.)	23.0 ±0.8 (n.s.)	100
THA 200μM（SOD-3/20℃）	21.6 ±0.5 (n.s.)	21.0 ±0.8 (n.s.)	100

n.s.= not significant

N = total worm number

P value compared to the Control (0) group

Table S4 （related to Figure5A, 5B）

Treatment	Mean Lifespan(d) ±SEM(P Value)	Median Lifespan(d) ±SEM(P Value)	N
Control（DAF-2/20℃）	52.2 ±1.5 (n.s.)	53.0 ±1.7 (n.s.)	95
EGCG 1000μM（DAF-2/20℃）	46.6 ±1.5 (<0.01)	47.0 ±1.2 (<0.01)	95
EGCG 1000μM + THA 200μM（DAF-2/20℃）	45.5 ±1.5 (<0.01)	45.0 ±1.6 (<0.01)	95
THA 200μM（DAF-2/20℃）	51.5 ±1.4 (n.s.)	53.0 ±1.8 (n.s.)	95
Control（DAF-16/20℃）	20.4 ±0.5 (n.s.)	21.0 ±0.5 (n.s.)	95
EGCG 1000μM（DAF-16/20℃）	18.9 ±0.5 (<0.01)	19.0 ±0.5 (<0.01)	95
EGCG 1000μM + THA 200μM（DAF-16/20℃）	19.1 ±0.5 (n.s.)	19.0 ±0.5 (n.s.)	95
THA 200μM（DAF-16/20℃）	20.6 ±0.5 (n.s.)	21.0 ±0.5 (n.s.)	96

n.s.= not significant

N = total worm number

P value compared to the Control (0) group

Figure S1 (related to Figure5D)

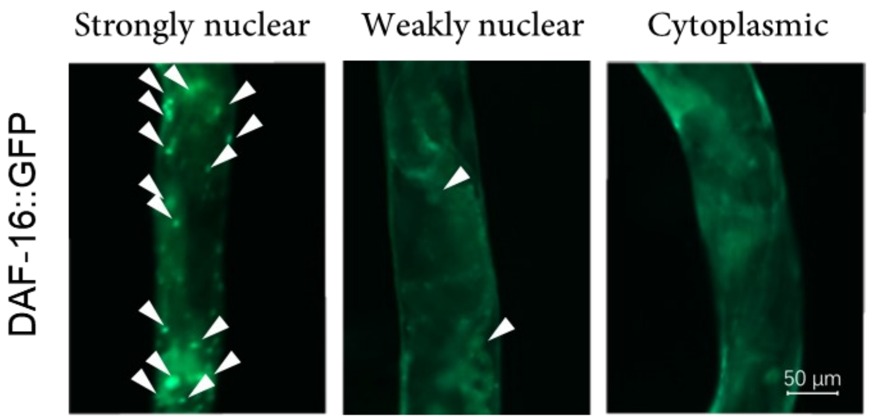

Figure S1 Representative images for DAF-16::GFP localization, each worm was given a score: cytoplasmic, 0; weakly nuclear, 1; strongly nuclear, 2.
